# Association of intensity and dominance of CEOs’ smiles with corporate performance

**DOI:** 10.1038/s41598-024-63956-2

**Published:** 2024-06-17

**Authors:** Ken Fujiwara, Pierrich Plusquellec

**Affiliations:** 1 Department of Psychology, National Chung Cheng University, Chiayi, 621301 Taiwan, ROC; 2 School of Psychoeducation, Université de Montréal, Montreal, H3T 1J4 Canada

**Keywords:** Human behaviour, Computer science

## Abstract

This study investigated whether the facial expressions of chief executive officers (CEOs) are associated with corporate performance. A photograph of the CEO or president of each company that appeared on the Fortune Global 500 list for 2018 was taken from the company’s official website. The smile intensity and action unit activation in each face were calculated using a pre-trained machine learning algorithm, FACET. The results revealed a positive association between smile intensity and company profit, even when controlling for the company’s geographic location (Western culture versus others) and the CEO’s gender. Furthermore, when the type of smile was examined with the activation of each action unit, this significant positive association was identified in the dominant smile but not in the reward and affiliative smiles. Relationships among the leader’s smile intensity, group strategy, and group performance are discussed.

## Introduction

Faces offer rich information about an individual’s characteristics, and facial expressions function as a major communication cue. Facial appearance forms much of a person’s first impression^1^ and indicates the individual’s race, age, gender^2^, sexual orientation^3^, and may also influence electoral outcomes^4,5^. Conversely, research on facial expressions has found that smile intensity (i.e., the extent of joy) in a photograph can indicate current life satisfaction^6^; it can even predict future prospects of marriage^7^, divorce^8^, longevity^9^, and political success^10^. Harker and Keltner^7^ suggested that positive emotionality in a posed smile could represent an individual’s social, cognitive, and behavioral repertoires. Cumulatively, these findings demonstrate that facial appearance and expressions can effectively embody aspects of an individual’s life.

What about groups? Previous studies have demonstrated that a group leader’s facial appearance can be representative of the group^11^. For example, Rule and Ambady^3,12^ found that perceptions (based on viewing facial expressions in photographs) of the leadership quality of chief executive officers (CEOs) could predict the company’s profit level, regardless of the CEO’s gender. However, the primary focus of Rule and Ambady’s studies was to assess the accuracy of naïve judgments, not the impact of the emotional expressions displayed on leaders’ faces. Similarly, Pillemer et al.^13^ investigated the association between the perceived traits through CEOs’ faces and their corporate performance, where the displayed positive affect was controlled for when assessing the CEO’s facial appearance. Hence, in examining the relationship between leaders’ faces and their firms’ performance, the affect on their faces has been treated as a variable to be controlled rather than a target.

Meanwhile, previous studies focusing on a leader’s facial expression have investigated its impact on followers’ performance^14,15^ or its appropriateness as a leader including perceived leadership^16,17^, not on group performance. To the best of our knowledge, Tsai et al.^18^ (Study 2b) is one of the limited studies that has directly examined the association between the leader’s smile and their group performance (i.e., company and university ranking). Although they did not find any significant association, it could be because the study placed its main focus on cultural rules of facial expressions rather than group success, and targeted only a limited sample from the United States and Taiwan/China (its impact will be discussed in the next section). Similarly, Harms et al.^19^, including the variable of positive emotional expression in their CEO’s facial impression measures, also failed to predict organizational outcomes perhaps because they only used the photographs of Chinese CEOs. Thus, the relationship between a leader’s facial expression and the objective performance of companies, if any, remains unclear.

In any investigation of leaders’ facial expressions, the display rule^20,21^ should be carefully addressed, as cultural rules governing the display of emotions^22^ might affect a leader’s facial expression. For example, Tsai et al.^18^ has indicated that leaders in Western countries such as the United States display more intense smiles than those from Eastern countries such as Taiwan and China, accurately representing their subordinates’ perception of an ideal emotional state in each case. For this reason, the variance in the intensity of a CEO’s smile might be harder to examine if all photos were collected from a single or a couple of countries, thus tending to reduce the range of facial expressions intensity. Indeed, Tsai et al.^18^ (Study 2b) did not find any clear relationships between the leader’s smile and their group performance. Another previous study collecting CEO’s facial photos only in China^19^ also failed to find any significant relations. Those non-significant results could be because the data were collected only from the United States and Taiwan, or China. In the present study, to cover a wider range of cultural styles, we used the Fortune Global 500 list and controlled for company location as a possible confounding factor.

Expanding the scope to the global leaders creates new problems. To assess fairly the facial expressions of CEOs from around the world, nonverbal dialects^23^ or in-group advantage^24,25^ must be addressed. Previous studies have suggested that it is difficult to judge the emotional expressions of individuals from other countries. Such a coding bias should offer valuable insights if the rating across cultures is itself the subject of focus. For instance, Harms et al.^19^ employed Western raters to evaluate the photographs of Chinese CEOs because it seemed valuable to test the efficacy of Western raters attempting to determine which CEOs are effective leaders in Eastern culture. However, the rater’s efficacy is not the primary focus of the current study. Rather, the coding bias could pose a serious problem; namely, using coders from only one country to judge the smile intensity of global leaders might yield unreliable results.

To overcome this issue and ensure the reliability of coding, other “eyes” will be needed. In that regard, computer vision and machine learning techniques offer an attractive and powerful alternative to ratings by human observers since they are less vulnerable to this cultural bias. Therefore, in this study, FACET 2.0 SDK from the iMotions software suite^26,27^ was employed. Furthermore, although machine learning techniques provide a high level of objectivity, their ratings of facial expressions could still be biased depending on the set of images on which they were trained^28^. Although whether this issue could indeed induce bias in the machine learning process remains an unresolved research question, at least, conservative ways should be preferred. Thus, to reduce this risk, an additional pre-trained machine learning algorithm, Face API (Microsoft), was employed to replicate the association between the global leaders’ smile intensity and their corporate performance.

In investigations of facial expressions, gender also should be considered. Many previous studies have shown that females tend to smile more than males^29–31^. Even among leaders, females show a greater preference for interpersonally oriented^32^ and participative leadership styles^33^, which would tend to result in a communal display (i.e., a clear smile). Indeed, in support of Eagly’s^34^ social role theory, Pillemer et al.^13^ illustrated that female CEOs were rated as high in supportive, compassionate, and warm, and such gender-linked traits from CEO’s faces, namely, higher powerfulness in male CEOs and higher supportiveness, compassion, and warmth in female CEOs, predicted better corporate performances. Therefore, as we predicted that female leaders would show stronger smiles than their male counterparts, we controlled for CEO gender when examining the relationship between CEOs’ smiles and corporate performance.

With so little prior research, it is unlikely that this study can narrow it down to a single prediction about the association between CEOs’ smiles and corporate performance. Thus, here proposes two competing predictions. First, it could be assumed that CEOs would display a strong smile if they were satisfied with or proud of their group’s high level of achievement (typically reflected by high company profits) since a smile is a common way of expressing pride^35^ and even dominance^36,37^. If this is true, the intensity of a CEO’s smile and corporate performance should have a positive association.

However, some previous studies focusing on smiles in interpersonal contexts have suggested the opposite association between smiles and performance–namely, that people with low levels of power or dominance smile more^38,39^. Whereas some studies reported no association^40^, Kraus and Chen^41^, who focused on a more interpersonally competitive situation (fighters facing their opponent), found a negative association between smiling and one’s performance during the match. Given that smiling is a submissive signal in competitive situations, one might hypothesize that the intensity of a CEO’s smile should be greater in companies earning lower profits. This study examines which of these two competing hypotheses is supported.

In addition to finding an association between the strength of a CEO’s smile and corporate performance, whether it is positive or negative, it is expected to offer one clue to determine the background rationale for the association by examining the type of smile displayed by the leader. As such, this study examines smiles in detail from two perspectives. One is the genuineness of the smile. Previous studies have distinguished a genuine smile with positive emotions (Duchenne smile) from an intentional smile (non-Duchenne smile)^42^. In this study, we focused on the activation of AU 6 (cheek raiser) and AU 12 (lip corner puller), which correspond to the movement of the orbicularis oculi (eye region) and the zygomatic major (mouth region), respectively^22^. The activation of AU 6 is combined with that of AU 12 to produce a genuine smile, whereas only AU 12 is intentionally activated in a non-Duchenne smile^42^. In this regard, we explore which of the AU activation in leaders’ faces, AU6 or AU 12, is more associated with corporate profit.

We also shed light on the functional perspective of smiles. Rychlowska et al.^37^ have identified different types of smiles with different functions: reward, affiliation, and dominance, to each of which different Action Units contribute. It is expected that if we know which function of smiles represents the association, we can better understand the underlying mechanism. Therefore, based on the aforementioned arguments, the hypotheses and exploratory research questions of this study are formulated as follows:

### H1a

The CEO’s smile intensity will be positively associated with corporate profit.

### H1b

The CEO’s smile intensity will be negatively associated with corporate profit.

### RQ1

Which of the Action Units, AU6 or AU 12, is more strongly associated with corporate profit?

### RQ2

Which type of functional smile, reward, affiliation, or dominance, is more associated with corporate profit?

## Results

Descriptive statistics and Pearson’s bivariate correlations of the smile measures are shown in Table 1. Note that the results of the principal component analysis (PCA) revealed that the first principal component explained 63.5%, 65.3%, and 59.3% of the total variance of reward, affiliative, and dominant smiles, respectively. Further, the results of the PCA replicated Rychlowska et al.^37^, indicating that affiliative smiles involved the Lip Pressor (AU24) and the Dimpler (AU14) in addition to the Lip Corner Puller (AU12). With respect to dominant smiles, higher levels of involvement by the Cheek Raiser (AU6) and the Nose Wrinkler (AU9) were detected, which also replicated Rychlowska et al.^37^ On the contrary, unlike Rychlowska et al.^37^, the Inner-Outer Brow Raiser (AUs 1 and 2) and the Dimpler did not contribute to the reward smiles in this study. See also Supplementary Results for descriptive statistics on corporate profits and the number of employees for each year (Table S1) and details of the principal component analysis (Table S2).

**Table 1:** *M*s and *SD*s of smile measures and their bivariate correlations.

	M	SD	1	2	3	4	5	6
FACET								
1. Smile intensity	2.337	2.46						
2. AU 6	0.880	1.236	0.828$$^{**}$$					
3. AU 12	1.177	1.381	0.949$$^{**}$$	0.890$$^{**}$$				
4. Reward	0.000	2.100	0.857$$^{**}$$	0.875$$^{**}$$	0.871$$^{**}$$			
5. Affiliative	0.000	1.696	0.845$$^{**}$$	0.879$$^{**}$$	0.892$$^{**}$$	0.958$$^{**}$$		
6. Dominant	0.000	1.611	0.565$$^{**}$$	0.809$$^{**}$$	0.629$$^{**}$$	0.734$$^{**}$$	0.654$$^{**}$$	
FaceAPI								
7. Smile intensity	0.718	0.389	0.729$$^{**}$$	0.648$$^{**}$$	0.687$$^{**}$$	0.654$$^{**}$$	00.602$$^{**}$$	00.610$$^{**}$$

*N* = 409 for FACET. *N* = 407 for FaceAPI. For Reward, Affiliative, and Dominant smiles, the first principal component score obtained from a principal component analysis was used. $$^{**}$$*p* < 0.01.

### Western culture versus others

We first considered whether CEOs’ smile intensity differed depending on the company’s location (Western versus Others). A *t*-test revealed that smile intensity was significantly greater among Western leaders ($n = 251$, $M = 3.09$, $SD = 2.20$) than among leaders from other countries such as China, Japan, and South Korea ($n = 158$, $M = 1.14$, $SD = 2.37$), $t(315.64) = 8.34$, $p <.001$, $d = 0.861$, 95% CI [0.653–1.069]. The same resulting pattern was found for AU 6, $M_\mathrm{{Western}} = 1.19$, $SD_\mathrm{{Western}} = 1.24$ versus $M_\mathrm{{Others}} = 0.39$, $SD_\mathrm{{Others}} = 1.05$, $t(372.84) = 9.95$, $p <.001$, $d = 0.680$, 95% CI [0.475–0.885] and for AU 12, $M_\mathrm{{Western}} = 1.57$, $SD_\mathrm{{Western}} = 1.37$ versus $M_\mathrm{{Others}} = 0.55$, $SD_\mathrm{{Others}} = 1.16$, $t(371.89) = 8.02$, $p <.001$, $d = 0.786$, 95% CI [0.579–0.993]. As for each type of smile, significant differences were also found: for reward smile, $M_\mathrm{{Western}} = 0.60$, $SD_\mathrm{{Western}} = 2.06$ versus $M_\mathrm{{Others}} = -0.95$, $SD_\mathrm{{Others}} = 1.78$, $t(368.85) = 8.07$, $p <.001$, $d = 0.792$, 95% CI [0.586–0.999]; for affiliative smile, $M_\mathrm{{Western}} = 0.35$, $SD_\mathrm{{Western}} = 1.77$ versus $M_\mathrm{{Others}} = -0.55$, $SD_\mathrm{{Others}} = 1.40$, $t(386.65) = 5.69$, $p <.001$, $d = 0.548$, 95% CI [0.346–0.751]; for dominant smile, $M_\mathrm{{Western}} = 0.49$, $SD_\mathrm{{Western}} = 1.45$ versus $M_\mathrm{{Others}} = -0.78$, $SD_\mathrm{{Others}} = 1.55$, $t(316) = 8.26$, $p <.001$, $d = 0.853$, 95% CI [0.645–1.060].

### Gender differences

Smile intensity was significantly greater among female leaders ($n = 13$, $M = 4.79$, $SD = 2.01$) than in their male counterparts ($n = 396$, $M = 2.26$, $SD = 2.43$), $t(13.18) = 4.45$, $p <.001$, $d = 1.048$, 95% CI [0.489–1.607]. The same was true for AU 6, $M_\mathrm{{Female}} = 2.14$, $SD_\mathrm{{Female}} = 1.32$ versus $M_\mathrm{{Male}} = 0.84$, $SD_\mathrm{{Male}} = 1.21$, $t(12.67) = 3.51$, $p <.001$, $d = 1.072$, 95% CI [0.513–1.631] and for AU 12, $M_\mathrm{{Female}} = 2.84$, $SD_\mathrm{{Female}} = 1.59$ versus $M_\mathrm{{Male}} = 1.12$, $SD_\mathrm{{Male}} = 1.34$, $t(12.57) = 3.85$, $p <.001$, $d = 1.272$, 95% CI [0.711–1.833]. As for each type of smile, significant differences were also found: for reward smile, $M_\mathrm{{Female}} = 1.76$, $SD_\mathrm{{Female}} = 1.89$ versus $M_\mathrm{{Male}} = -0.06$, $SD_\mathrm{{Male}} = 2.08$, $t(12.97) = 3.39$, $p <.001$, $d = 0.874$, 95% CI [0.319–1.430]; for affiliation smile, $M_\mathrm{{Female}} = 1.78$, $SD_\mathrm{{Female}} = 1.89$ versus $M_\mathrm{{Male}} = -0.06$, $SD_\mathrm{{Male}} = 1.66$, $t(12.62) = 3.47$, $p <.001$, $d = 1.104$, 95% CI [0.547–1.662]. However, such a gender difference was not significant for the dominant smile, $M_\mathrm{{Female}} = 0.72$, $SD_\mathrm{{Female}} = 1.62$ versus $M_\mathrm{{Male}} = -0.02$, $SD_\mathrm{{Male}} = 1.61$, $t(12.79) = 1.62$, $p =.130$, $d = 0.460$, 95% CI [$-0.093$–1.014].

### Associations between smiles and corporate performance

**Figure 1:**
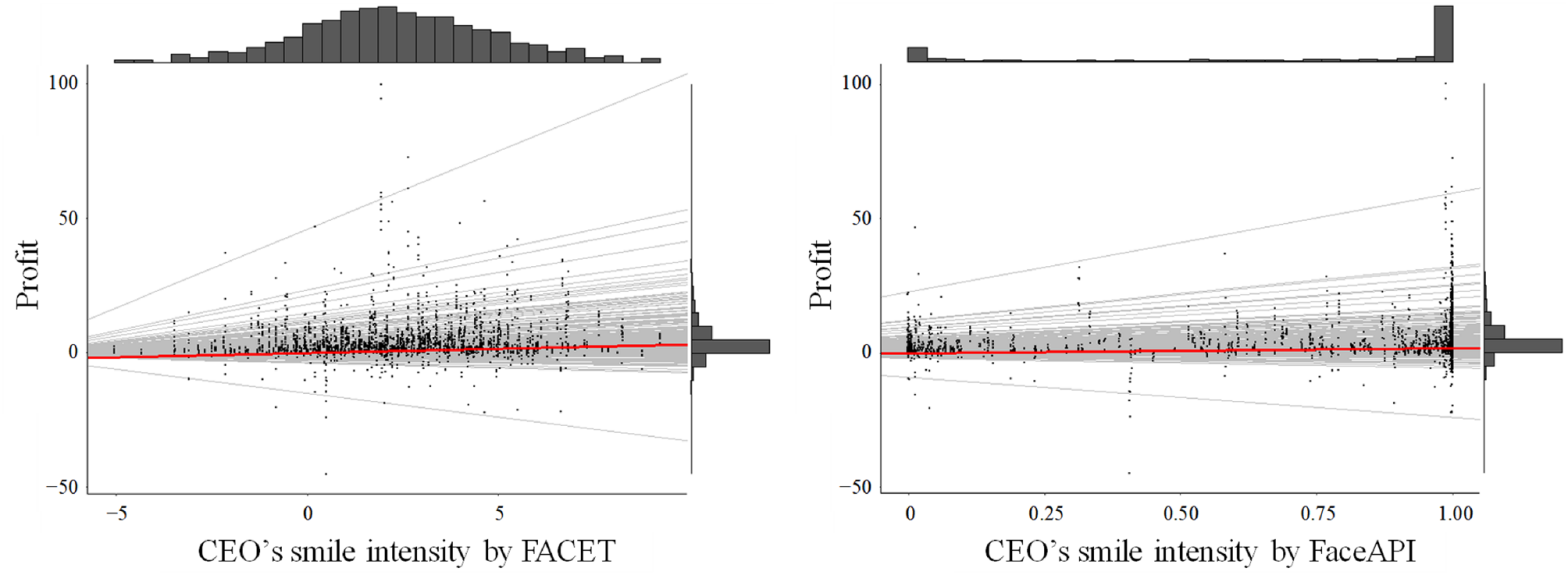
Scatterplots of corporate profit and CEOs’ smile intensity as calculated by FACET (left) and Face API (right). The gray lines represent the random intercept and slope for the smile intensity, whereas the red line represents the average intercept and slope.

We ran an HLM on the 8-year corporate profits, controlling for the company’s location, leader gender, and number of employees, and adjusting for degrees of freedom in the nested data structure (Table 2). The result indicated a significant positive association between the leader’s smile intensity and the company profit, which was replicated with data from the Face API (Figure 1). Regarding the activation of AUs 6 and 12, only AU 12 yielded a significant positive association with corporate profit. For each type of smile, a significant positive association was found only for the dominant smile, but not for the reward and affiliation smiles (Table 2). Note that the resulting patterns were more pronounced when analyzing past profit (fiscal years 2015–2018), but not so for future profit (fiscal years 2019–2022), see Supplementary Results.

## Discussion

Previous studies have demonstrated that a leader’s facial appearance could represent their group performance^3,12^. However, this study provided the first evidence that a leader’s facial expression can be an embodiment of the group. First, as predicted, we demonstrated that the CEO’s smile intensity differed depending on the company’s geographic location, supporting previous findings that a leader’s facial expression reflects an aspect of cultural norms^18^. Likewise, the female CEOs showed a greater smile than their male counterparts, consistent with previous studies that reported a gender difference in the display of smiles^29–31^. Notably, the gender difference was more salient in the reward and affiliative smiles but not in the dominant smile. Furthermore, the finding on the smile intensity was confirmed by two different facial expression analysis algorithms based on recent machine learning techniques, an approach that overcomes the possible decoding bias in human observers^23–25^ and hence possesses a higher reliability. Yet, it is also true that machines are not bias-free but have their own biases depending on the set of images on which they were trained.^28^

We also found that CEOs’ smile intensity was positively associated with corporate performance, which supports H1a. Although the association may not be so strong (Figure 1), it is nevertheless impressive, considering that we employed publicly available, objective data. Furthermore, the association remained significant even when we controlled for the possible confounding factors of the company’s location and the CEO’s gender. However, it should be noted that the impact of the COVID-19 pandemic on the economy cannot be ignored. Since the fiscal year 2020, many companies have irregularly lost their profit due to the unpredictable global disaster. For example, many companies in certain industries, such as airlines, disappeared from the Global 500 ranking, which may have unexpectedly distorted the results. In fact, the association between CEO smile intensity and corporate profit weakened after fiscal year 2020 when future profit and profit in each fiscal year were analyzed (Table S6). Thus, we may need to be aware that the leader’s facial expression does not predict group performance in an emergency.

The interpersonal power account (H1b) cannot explain this positive association because it interprets a smile in competitive circumstances as a submissive signal^41^. Clearly, for leaders, smiling is not a display of submissiveness. As we examine what kind of smile the leaders had on their faces, AU 12 was significantly associated with corporate performance but not AU 6, which implies that a strategic component of CEOs’ facial display played a role (RQ1). Smiles with only AU 12 activated are non-Duchenne smiles, i.e., not a genuine expression but a controllable social signal^42,43^. Companies and their CEOs would make strategic decisions on which photos to display on their website (even if the photo may have been taken a long time ago, the decision to put it on the website is current), and their selection appears to have been associated with company financial performance.

In terms of the smile function^37,44^, the results showed that a significant positive association was identified in the dominant smile but not in the reward and affiliative smiles (RQ2). This implies that a CEO’s smile indicates their dominance over competitive groups, which is the opposite of what occurs in an interpersonally competitive context^41^. Another possibility is that the smiling CEOs were focused on their intragroup membership (i.e., their relationships with their employees) since a leader with a smile is perceived as more suitable for leadership than one with a nervous expression^17^. Unfortunately, the current study cannot examine these interesting possibilities since we do not know which strategies the companies actually applied. However, at least, the CEO’s smile does not seem to be a direct expression of their desire to express a sense of affiliation with their customers since smiles are perceived as warmer but as indications of less competence by customers in a marketing context^45^. In the future, the group strategy (i.e., whether the primary target audience is within or outside the group) should be included when investigating the relationship between the leader’s smile intensity and the group’s performance. Such a study should contribute to the development of new theoretical accounts of leader’s facial expressions.

As a limitation, the current study could not identify the temporal relation; we could not determine exactly when the CEO’s facial photo was taken and uploaded. The leaders may have displayed a dominant smile in response to the outstanding performance of the company, however, the group inspired by such a leader could be more successful in its performance. It may be unlikely that major global companies would change the CEO’s picture on their website every year, which means the group performance is not immediately reflected in the leader’s expression. The constant and longitudinal data collection required to determine temporal causality is also a future research task. A longitudinal study would also enable researchers to test if smile intensity can predict future “life” characteristics, observed at an individual level^7–9^, in a group context. This possibility was investigated in this study, however, unfortunately, the results were unstable and inconclusive because the current study has only four fiscal years in the future profit data, and they seemed to be greatly influenced by the COVID-19 pandemic. With the aid of the recent development of computer vision and machine learning techniques, researchers are now able to efficiently perform objective and reliable facial expression coding. The predictive impact of a leader’s smile on group performance will be fairly investigated in the future.

Another limitation can be attributed to the characteristics of the list of companies included in this study. The CEO photographs selected from the Fortune Global 500 companies may not represent typical corporate leadership. Such global companies may engage in specific branding and image management practices that are not common in smaller companies. Therefore, the findings may not be generalizable to smaller firms or different sectors. Relatedly, the influence of gender norms on the perception and effectiveness of leaders’ smiles should be further explored, as this study cannot fully address the influence of gender norms due to sampling bias with respect to gender (i.e., the small number of female CEOs). For example, we cannot rule out the possibility that the CEO’s dominant smile was associated with profits and reward or affiliative smiles were not because the primary population of the CEO sample was male. To address these concerns, one possible approach would be to target companies of different sizes or groups of different disciplines (e.g., sports athletes). Other experimental studies, such as group assignments with leaders and followers in a laboratory setting, would also offer fruitful implications.

## Methods

### Targets, sample size, and its justification

In this study, only publicly available data were used for the analysis. We used the Fortune Global 500 database to generate our sample (https://fortune.com/global500/). Each company’s annual objective performance was assessed on the basis of its profit (in millions of dollars) for fiscal year 2015–2022 obtained from the database. The number of employees was also treated as a control variable since a company’s performance might depend on its size.

A photograph of each Fortune Global 500 company’s CEO or president (some Asian companies do not have a CEO) was taken from the company’s official website in February 2019. As for the company list, the Fortune Global 500 of 2018 (Fiscal Year 2017) was used. If the photo included other board members as well as the leader, we cropped out the leader’s face. Some company websites did not display a photograph of their leader, and some photos were too small to permit sufficiently precise extraction of facial expressions using our two machine learning algorithms. As a result, the sample consisted of 409 photos for the FACET algorithm (female = 13, male = 396) and 407 for Face API (female = 14, male = 393).

The list of these companies was used to extract the corporate profits for other years. However, due to the nature of the list, the targeted companies could be missing from the list in some years. With this in mind, the sample sizes available for analysis (and the ratio of missing values to the reference value of 409, the number of CEO smiles obtained from the 2018 list) were as follows: 373 in 2015 (8.8%), 387 in 2016 (5.3%), 409 in 2017, 390 in 2018 (4.6%), 378 in 2019 (7.6%), 358 in 2020 (12.4%), 347 in 2021 (15.2%), and 328 in 2022 (19.8%). See also Table S1. The total number of data points included in the HLM was 2865 (FACET) and 2947 (FaceAPI) for a total of 8 years, 1506 (FACET) and 1547 (FaceAPI) for the past 4 years, and 1359 (FACET) and 1400 (FaceAPI) for the future 4 years.

**Table 2:** Culture/location: western $= 1$, others $= 0$.

	Corporate profit													
	*b*	*SE*	*b*	*SE*	*b*	*SE*	*b*	*SE*	*b*	*SE*	*b*	*SE*	*b*	*SE*
Fixed effect														
Intercept	1.70$$^{**}$$	0.44	1.87$$^{**}$$	0.45	1.69$$^{**}$$	0.45	2.22$$^{**}$$	0.53	2.19$$^{**}$$	0.51	2.17$$^{**}$$	0.48	1.32$$^{**}$$	0.40
Num. employees	7.18$$^{**}$$	1.37	7.02$$^{**}$$	1.34	7.22$$^{**}$$	1.37	7.16$$^{**}$$	1.32	7.67$$^{**}$$	1.35	6.69$$^{**}$$	1.28	6.05$$^{**}$$	1.14
Culture/Location	0.94	0.60	1.28	0.56	1.08	0.58	1.24	0.64	1.26$$^{*}$$	0.60	1.48$$^{**}$$	0.51	0.72	0.53
Gender	$-1.62$	1.94	$-1.25$	1.91	$-1.72$	1.99	$-0.89$	1.61	$-1.08$	1.52	$-0.13$	1.64	$-0.73$	1.74
FACET Smile intensity	0.31$$^{**}$$	0.12												
FACET AU 6			0.42	0.23										
FACET AU 12					0.58$^{*}$	0.23								
FACET Reward							0.13	0.15						
FACET Affiliative									0.19	0.32				
FACET dominant											0.36$$^{**}$$	0.14		
FaceAPI smile intensity													1.90$$^{**}$$	0.61

	Variance		Variance		Variance		Variance		Variance		Variance		Variance	
Random effect														
Intercept	18.06		20.99		18.89		30.11		23.04		30.27		5.74	
Slope	0.28		1.16		0.98		0.003		2.69		1.33		15.05	
Residual	20.34		20.35		20.34		20.35		20.35		20.32		19.99	

Gender: female $$= 1$$, Male $$= 0$$. Weights in Table 2 are unstandardized but profit and the number of employees are rescaled divided by 1000 and 1,000,000, respectively, for better visibility. $$^{*}$$*p* < 0.05, $$^{**}$$*p* < 0.01.

The sample size of this study is larger than Tsai et al.^18^ (Study 2b) that examined the smile intensity of the U.S. and Taiwanese/Chinese leaders (*N* = 170, 112, respectively) and Harms et al.^19^ of 71 Chinese CEOs. Besides, in the previous studies, the variance in the smile intensity could be unfairly reduced since the data were collected only from the United States and Taiwan/China, which might result in a decreased power compared to the original sample size. We also used eight years of firm performance data in our analysis rather than a single year, which is expected to find a stable association. Thus, this study should be more powerful than the previous study to detect a significant association between the CEO’s smile intensity and corporate performance.

### Quantifying smile intensity and the activation of action units

FACET automatically calculated smile intensity (or indication of the degree of joy) and the activation of specific Action Units (AUs), or individual components of facial muscle movement. The FACET output was represented as the odds, on a logarithmic (base 10) scale, that a target expression was present. For instance, a smile intensity of 1 indicated that the observed expression was 10 times more likely to be categorized by an expert human coder as joyful than as not joyful. We used the same scoring procedure for the AU measures, represented as the odds of the activation of the target AU on a logarithmic (base 10) scale.

To investigate RQ1, the activation of AUs 6 (cheek raiser) and 12 (lip corner puller) are used. For RQ2, following the previous studies^37,44^, the activation of different AUs was used to estimate the extent of different types of smiles. The reward smile includes the activation of AUs 1 (Inner Brow Raiser), 2 (Outer Brow Raiser), 10 (Upper Lip Raiser), 12, 14 (Dimpler), and 25 (Lips part). The affiliation smile includes the activation in AUs 10, 12, 14, and 24 (Lip Pressor). The dominance smile involves the activation of AUs 5 (Upper Lid Raiser), 6, 9 (Nose Wrinkler), and 10. For the degree of each smile, we used the first principal component score obtained from a principal component analysis using the target AUs. Please note that, according to Rychlowska et al.^37^, dominant smiles include the unilateral right or left Lip Corner Puller (AU12R or AU12L). Because the FACET software does not provide the measure for asymmetric displays, we excluded AU 12 from the principal component analysis for dominant smiles. It should be noted that the significance of the results remained the same when AU 12 was included for the dominant smiles.

Additionally, for the smile intensity measure, a second algorithm (Face API, Microsoft) was employed to limit the risk of bias resulting from a specific algorithm. Face API provides a different type of output from FACET, calculating the confidence level of seven emotional components–happiness, sadness, anger, surprise, disgust, fear, and neutral–with scores that must add to a total of 1 (see https://azure.microsoft.com/en-us/services/cognitive-services/face/). We used the happiness score awarded by Face API (also called the smile score in the algorithm) as our second measure of smile intensity. Note that the Face API smile intensity does not appear to be normally distributed (Figure 1). However, we used this measure as it is because it is an explanatory variable rather than a respondent variable in the model, and it is a secondary variable, used collectively to test our hypothesis along with the smile intensity by FACET.

### Control variables

#### Culture/location

As shown in Tsai et al.^18^, CEOs’ smile intensity would differ depending on the company’s geographic location, with CEOs from Western companies displaying a greater intensity than leaders from other cultures. Thus, we controlled for company location as a possible confounding factor: i.e., a binary dummy code; Western (North America, Western Europe, and Australia) $= 1$, Others (Asia and South America) $= 0$.

#### CEO gender

As previous studies have shown that females tend to smile more than males^29–31^, we predicted that female leaders would indicate a clear smile. Thus, as with company location, we controlled for CEO gender (i.e., a binary dummy code; Female $= 1$, Male $= 0$).

### Analysis strategy

The measured variables in this study were on varying levels. The CEO’s smile intensity, the company’s geographic location, and CEO gender measures were on the company level (level 2), whereas the corporate profit and number of employees were on the year level (level 1). The four years prior to the collection of the photos (i.e., fiscal years 2015–2018) could be considered as measures of the corporate past profit, while fiscal years 2019–2022 represent the corporate future profit. However, since we were unable to identify the exact dates when the photos were published and when the photo was subsequently changed, a strict causal relationship between the corporate profit and the CEO’s smile cannot be established. Instead, all eight years of profit data obtained were used in the analysis (see Tables S4 and S5 in Supplementary Results for a separate analysis of the past and future profit).

Because of the nested structure of the data, the study applied hierarchical linear modeling (HLM). Given the structure of the data, in which the profits of the target companies are collected over 8 years, two grouping factors were considered for the random effects: one for the company and the other for the year. The intraclass correlation coefficient (*ICC*) was calculated for each grouping variable, indicating that $ICC = 0.603$ for companies, while $ICC = 0.033$ for years. Since only 3.3% of the total variance exists at the year level, the year was not included as a random effect in the model. For the company random effect, we calculated the Akaike Information Criterion (AIC), an indicator of the goodness of model fit, to determine whether to include a random slope in addition to the random intercept. Because the AIC was lower for the random slope model in all models except when the reward was used as the smile measure (Table S3), we included a random slope for the measurement of the smile in the final model. The model was formulated using R’s lmer4 syntax as follows:

$$\begin{aligned} Profit = b0 + b1*Num.employee + b2 *Culture/Location + b3*Gender + b4* SMILE + (SMILE|Company) \end{aligned}$$

where SMILE refers to one of the smile measurements (i.e., the intensity of the smile, the activation of AUs 6 and 12, and the degree of reward, affiliation, or dominance). Note that each of the CEO smile variables was entered separately, and we reported the model with a random slope even when the reward was used as the smile variable for comparison.

### Supplementary Information

Supplementary Information.

## Data Availability

Data reported here as well as code analyzing data is available at the OSF website (https://osf.io/zu439).
