# Supplementary material for: Association of intensity and dominance of CEOs’ smiles with corporate performance
Source: Sci Rep. 2024 Jun 17;14:13986. doi: 10.1038/s41598-024-63956-2 (PMC11183120; doi:10.1038/s41598-024-63956-2)
Supplement: Supplementary file 1 — Supplementary Information. [file 41598_2024_63956_MOESM1_ESM.pdf]

Association of intensity and dominance of CEOs' smiles with corporate performance

Supplementary results

Table S1 *M*s and *SD*s of Corporate Profit and the Number of Employees in Each Fiscal Year

		Profit		Employees	
Fiscal Year	*n*	*M*	*SD*	*M*	*SD*
2015	373	2978.681	5868.076	138657	185142
2016	387	3006.683	4777.654	136916	180688
2017	409	3730.369	5926.058	134855	177702
2018	390	4217.582	6239.358	140525	176485
2019	378	3889.355	6241.866	142226	179141
2020	358	3129.563	7410.072	149284	190553
2021	347	6355.032	9262.884	150812	196019
2022	328	6318.064	10819.115	155303	193594

Note. Profits: Million USD. Values represent companies for which a CEO's photo was available, as analyzed by FACET.

Table S2 Principal Component Analysis for Reward, Affiliative, and Dominant Smiles

	Comp.1	Comp.2	Comp.3	Comp.4	Comp.5	Comp.6
Reward						
AU 1	.223	.644	.185	.177	.103	.677
AU 2	.204	.585	.224	.192	−.123	−.717
AU 10	−.163	−.425	.887			
AU 12	−.573	−.130	.765	.249		
AU 14	.264	−.200	.203	.168	.896	-.133
AU 25	−.696	.425	−.329	−.235	.405	
Proportion of Variance	.634	.154	.101	.069	.033	.008
Cumulative Proportion	.634	.789	.889	.959	.992	1.000
Affiliative						
	Comp.1	Comp.2	Comp.3	Comp.4		
AU 10	.163	.134	.969	.129		
AU 12	.726	.654	−.211			
AU 14	−.359	.379	−.105	.846		
AU 24	−.564	.640	−.516			
Proportion of Variance	.652	.194	.129	.025		
Cumulative Proportion	.652	.846	.975	1.000		
Dominant						
	Comp.1	Comp.2	Comp.3	Comp.4		
AU 5	.205	.423	.220	.855		
AU 6	−.621	−.638	.454			
AU 9	−.673	.604	−.425			
AU 10	−.346	.220	.877	−.251		
Proportion of Variance	.596	.264	.073	.066		
Cumulative Proportion	.596	.861	.934	1.000		

Note. The 'princomp' function in R was used for the principal component analysis. Reward and Dominant smile were inverted by multiplying by −1 when used in subsequent statistical analyses, as the benchmark AUs (6 and 12) were negatively loaded, respectively.

Table S3 Summary of AIC

	Model	
Smile variable	Random intercept	Random slope
FACET Smile intensity	17735.08	**<u>17723.38</u>**
FACET AU 6	17737.49	**<u>17729.23</u>**
FACET AU 12	17735.45	**<u>17725.55</u>**
FACET Reward	**<u>17737.99</u>**	17741.70
FACET Affiliative	17737.66	**<u>17730.55</u>**
FACET Dominant	17737.47	**<u>17702.87</u>**
FaceAPI Smile intensity	18187.44	**<u>18133.41</u>**

Note. The lower the AIC, the better the model fits the data.

The following is the formula for each model written our using the R, lmer4 syntax.

Random intercept model:

Profit = b0 + b1*Num.employee + b2 *Culture/Location + b3*Gender + b4* SMILE + (1|Company)

Random slope model:

Profit = b0 + b1*Num.employee + b2 *Culture/Location + b3*Gender + b4* SMILE + (SMILE|Company)

where SMILE refers to either of the smile measurements (i.e., the intensity of the smile, the activation of AUs 6 and 12, and the degree of reward, affiliative, or dominance). Note that we used the argument "REML = FALSE" to compare models using AIC, since maximum likelihood, ML (not restricted maximum likelihood, REML), does not depend on the coefficients of the fixed effects. Setting the argument to "REML = TRUE" did not change the resulting pattern.

Table S4 Association Between the CEO's Smile and Corporate Past Profit (Fiscal Year 2015–2018)

	Corporate profit													
	b	*SE*	*b*	*SE*	*b*	*SE*	*b*	*SE*	*b*	*SE*	*b*	*SE*	*b*	*SE*
Fixed effect														
Intercept	**1.69****	0.39	**1.97****	0.40	**1.72****	0.39	**2.16****	0.49	**2.14****	0.48	**2.37****	0.44	**1.41****	0.38
Num. employees	**4.99****	1.40	**4.48****	1.35	**4.81****	1.39	**5.24****	1.42	**5.50****	1.42	**4.29****	1.30	**3.71****	1.15
Culture/Location	0.66	0.53	**0.98***	0.49	0.79	0.50	1.04	0.56	1.01	0.55	**0.98****	0.45	0.39	0.49
Gender	−2.22	1.76	−1.94	1.72	−2.44	1.82	−1.40	1.53	−1.51	1.48	−0.63	1.46	−1.18	1.55
FACET Smile intensity	**0.33****	0.10												
FACET AU 6			**0.41***	0.20										
FACET AU 12					**0.61***	0.20								
FACET Reward							0.16	0.14						
FACET Affiliative									0.21	0.17				
FACET Dominant											**0.39****	0.12		
FaceAPI Smile intensity													**1.85****	0.56
Random effect														
	Variance		Variance		Variance		Variance		Variance		Variance		Variance	
Intercept	13.64		15.60		13.66		24.27		22.82		23.92		5.65	
Slope	0.27		1.25		1.11		0.02		0.52		1.07		10.45	
Residual	8.47		8.48		8.47		8.49		8.49		8.46		8.36	

Note. Culture/Location: Western = 1, Others = 0. Gender: Female = 1, Male = 0. Weights in Table S4 are unstandardized but profit and the number of employees are rescaled divided by 1,000 and 1,000,000, respectively, for better visibility. $^{*}p < .05$, $^{**}p < .01$

Table S5 Association Between the CEO's Smile and Corporate Future Profit (Fiscal Year 2019–2022)

	Corporate profit													
	b	*SE*	*b*	*SE*	*b*	*SE*	*b*	*SE*	*b*	*SE*	*b*	*SE*	*b*	*SE*
Fixed effect														
Intercept	**2.04****	0.60	**2.17****	0.62	**2.02****	0.61	**2.62****	0.67	**2.57****	0.64	**2.42****	0.64	**1.50****	0.49
Num. employees	**7.32****	1.99	**7.41****	1.93	**7.45****	1.99	**7.03****	1.77	**7.64****	1.78	**7.23****	1.81	**6.68****	1.58
Culture/Location	1.38	0.78	1.69	0.75	1.49	0.77	**1.60***	0.81	**1.65***	0.75	**1.92****	0.68	1.13	0.65
Gender	−1.07	2.33	−0.67	2.28	−1.12	2.36	−0.46	1.91	−0.74	1.78	0.23	2.07	−0.36	2.16
FACET Smile intensity	0.30	0.16												
FACET AU 6			0.41	0.31										
FACET AU 12					0.55	0.30								
FACET Reward							0.11	0.18						
FACET Affiliative									0.17	0.23				
FACET Dominant											0.37	0.19		
FaceAPI Smile intensity													**2.02****	0.76
Random effect														
	Variance		Variance		Variance		Variance		Variance		Variance		Variance	
Intercept	27.45		32.03		29.68		41.47		30.23		42.38		4.04	
Slope	0.27		0.90		0.77		0.06		4.22		1.97		30.49	
Residual	29.23		29.24		29.23		29.25		29.26		29.06		28.66	

Note. Culture/Location: Western = 1, Others = 0. Gender: Female = 1, Male = 0. Weights in Table S5 are unstandardized but profit and the number of employees are rescaled divided by 1,000 and 1,000,000, respectively, for better visibility. $^{*}p < .05$, $^{**}p < .01$

Table S6 Association Between the CEO's Smile and Corporate Profit in Each Fiscal Year

	Profit															
	2015		2016		2017		2018		2019		2020		2021		2022	
	β	*SE*	β	*SE*	β	*SE*	β	*SE*	β	*SE*	β	*SE*	β	*SE*	β	*SE*
FACET																
Num. employees	**.18****	.05	**.20****	.05	**.13****	.05	**.13***	.05	**.20****	.05	**.16***	.05	**.18***	.05	.10	.06
Culture/Location	.05	.06	.03	.05	.05	.05	.08	.05	.10	.05	−.04	.06	.15	.05	**.14***	.06
Gender	−.03	.05	−.02	.05	−.08	.05	−.06	.05	−.04	.05	−.02	.05	−.06	.06	.02	.05
Smile intensity	**.12***	.06	**.13***	.06	**.11***	.05	.08	.06	.10	.05	.10	.06	.05	.05	.01	.06
R^2	**.05****		**.06****		**.04****		**.03****		**.07****		**.04****		**.07****		**.03***	
Face API																
Num. employees	**.18****	.05	**.20****	.05	**.14****	.05	**.13****	.05	**.20****	.05	**.17****	.05	**.18****	.05	**.11***	.05
Culture/Location	.03	.06	.02	.06	.04	.06	.07	.06	.09	.06	−.04	.06	.14	.06	**.12***	.06
Gender	−.03	.05	−.02	.05	−.08	.05	−.06	.05	−.04	.05	−.02	.05	−.06	.05	.02	.05
Smile intensity	**.16****	.06	**.13***	.06	**.14***	.06	**.11***	.06	**.15****	.06	.10	.06	.11	.06	.08	.06
R^2	**.06****		**.06****		**.04****		**.04****		**.08****		**.04****		**.08****		**.04****	

Note. Culture/Location: Western = 1, Others = 0. Gender: Female = 1, Male = 0. Weights in Table S6 are standardized. $^{*}p < .05$, $^{**}p < .01$

Table S7 Association Between the CEO’s Smile of AUs 6 and 12 and Corporate Profit in Each Fiscal Year

	Profit															
	2015		2016		2017		2018		2019		2020		2021		2022	
	β	*SE*	β	*SE*	β	*SE*	β	*SE*	β	*SE*	β	*SE*	β	*SE*	β	*SE*
FACET																
Num. employees	**.18****	.05	**.20****	.05	**.13****	.05	**.13***	.05	**.20****	.05	**.17***	.05	**.18***	.05	.10	.05
Culture/Location	.07	.05	.06	.05	.08	.05	.09	.05	**.12***	.05	−.01	.06	**.17***	.06	**.15***	.06
Gender	−.03	.05	−.01	.05	−.07	.05	−.05	.05	−.04	.05	−.01	.05	−.05	.05	.02	.05
AU 6	.08	.05	.07	.05	.04	.05	.05	.06	.08	.05	.02	.06	.01	.06	.003	.06
AU 12																
R^2	**.05****		**.05****		**.03***		**.03***		**.06****		**.03***		**.06****		**.03***	
FACET																
Num. employees	**.18****	.05	**.20****	.05	**.13****	.05	**.13****	.05	**.20****	.05	**.17****	.05	**.18****	.05	.10	.05
Culture/Location	.06	.06	.03	.06	.06	.05	.08	.06	.10	.05	−.03	.06	**.16****	.06	**.14***	.06
Gender	−.04	.05	−.03	.05	−.08	.05	−.06	.05	−.05	.05	−.02	.05	−.06	.05	.02	.05
AU 6																
AU 12	**.11***	.06	**.12***	.06	.09	.06	.08	.06	**.11***	.05	.07	.06	.05	.06	.01	.06
R^2	**.05****		**.06****		**.04****		**.03****		**.07****		**.03***		**.07****		**.03***	

Note. Culture/Location: Western = 1, Others = 0. Gender: Female = 1, Male = 0. Weights in Table S7 are standardized. $^{*}p < .05$, $^{**}p < .01$

Table S8 Association Between the CEO's Smile in Each Function and Corporate Profit in Each Fiscal Year

	Profit															
	2015		2016		2017		2018		2019		2020		2021		2022	
	β	*SE*	β	*SE*	β	*SE*	β	*SE*	β	*SE*	β	*SE*	β	*SE*	β	*SE*
FACET																
Num. employees	**.18****	.05	**.20****	.05	**.13****	.05	**.13***	.05	**.20****	.05	**.17****	.05	**.18****	.05	.10	.06
Culture/Location	.08	.06	.05	.05	.08	.05	.09	.05	**.12***	.05	−.02	.06	**.17****	.06	**.15***	.06
Gender	−.02	.05	−.01	.05	−.07	.05	−.05	.05	−.04	.05	−.01	.05	−.05	.05	.02	.05
Reward	.06	.06	.07	.05	.04	.05	.05	.06	.07	.05	.04	.06	.01	.06	−.01	.06
Affiliative																
Dominant																
R^2	**.04****		**.05****		**.03***		**.03***		**.06****		**.03***		**.06****		**.03***	
FACET																
Num. employees	**.18****	.05	**.20****	.05	**.13****	.05	**.13****	.05	**.20****	.05	**.17****	.05	**.18****	.05	.10	.05
Culture/Location	.08	.06	.06	.05	.08	.05	.09	.05	**.12***	.05	−.02	.05	**.17****	.05	**.15***	.06
Gender	−.03	.05	−.02	.05	−.07	.05	−.06	.05	−.04	.05	−.02	.05	−.05	.05	.02	.05
Reward																
Affiliative	.07	.05	.08	.05	.06	.05	.06	05	.08	.05	.06	.06	.01	.06	−.02	.06
Dominant																
R^2	**.05****		**.05****		**.03***		**.03****		**.06****		**.03***		**.06****		**.03***	

Table S8 (continued) Association Between the CEO's Smile in Each Function and Corporate Profit in Each Fiscal Year

	Profit															
	2015		2016		2017		2018		2019		2020		2021		2022	
	β	*SE*	β	*SE*	β	*SE*	β	*SE*	β	*SE*	β	*SE*	β	*SE*	β	*SE*
FACET																
Num. employees	**.18****	.05	**.20****	.05	**.13****	.05	**.13***	.05	**.20****	.05	**.17***	.05	**.18***	.05	.10	.05
Culture/Location	.07	.06	.06	.05	.08	.05	.08	.05	**.11***	.05	−.01	.06	**.17***	.06	**.13***	.06
Gender	−.02	.05	−.01	.05	−.07	.05	−.05	.05	−.03	.05	−.01	.05	−.05	.05	.02	.05
Reward																
Affiliative																
Dominant	.07	.06	.05	.05	.04	.05	.08	.05	.07	.05	.02	.06	.02	.06	.05	.06
R^2	**.05****		**.05****		**.03***		**.03***		**.06****		**.03***		**.06****		**.04***	

Note. Culture/Location: Western = 1, Others = 0. Gender: Female = 1, Male = 0. Weights in Table S8 are standardized. $^{*}p < .05$, $^{**}p < .01$
